# Social Anxiety in Children and Adolescents With Autism Spectrum Disorders Contribute to Impairments in Social Communication and Social Motivation

**DOI:** 10.3389/fpsyt.2020.00710

**Published:** 2020-07-24

**Authors:** Kellen Briot, François Jean, Ali Jouni, Marie-Maude Geoffray, Myriam Ly-Le Moal, Daniel Umbricht, Christopher Chatham, Lorraine Murtagh, Richard Delorme, Manuel Bouvard, Marion Leboyer, Anouck Amestoy

**Affiliations:** ^1^ University of Bordeaux, Medical Sciences Department, Bordeaux, France; ^2^ Centre Hospitalier Charles-Perrens, Pôle Universitaire de Psychiatrie de l’Enfant et de l’Adolescent, Bordeaux, France; ^3^ Centre Hospitalier Dr Jean Eric Techer, Pôle de Psychiatrie de l'enfant et de l'adolescent, Calais, France; ^4^ Department of Child and Adolescent Psychiatry, Centre Hospitalier le Vinatier, Bron, France; ^5^ Institut Roche, Tour Horizons- Bureau 18M3, Boulogne-Billancourt, France; ^6^ Roche Pharma Research and Early Development, Roche Innovation Center Basel, F. Hoffmann-La Roche Ltd., Basel, Switzerland; ^7^ Fondation FondaMental, Créteil, France; ^8^ Institut Pasteur, Human Genetics and Cognitive Functions Unit, Paris, France; ^9^ Université Paris Est Créteil, AP-HP, DMU IMPACT, Psychiatry and Addictology Department, Mondor University Hospital, Créteil, France; ^10^ Aquitaine Institute for Cognitive and Integrative Neuroscience, INCIA UMR 5287, Bordeaux, France; ^11^ INSERM, U955, IMRB, Laboratoire de NeuroPsychiatrie translationnelle, Créteil, France

**Keywords:** autism spectrum disorders, child, adolescent, social anxiety, comorbidity, prevalence

## Abstract

**Background:**

Recognition of symptoms of Social anxiety (SA) may be difficult among individuals with Autism Spectrum Disorders (ASD) because of overlap between social anxiety and autistic symptomatology. The main aim of our study was thus to explore the association between symptoms of social anxiety and clinical characteristics of ASD in order to identify individuals experiencing concomitant ASD and social anxiety disorder. We also described the prevalence of SA in a sample of children and adolescents with ASD.

**Method:**

79 children and adolescents with ASD (with and without intellectual disability) and 28-matched control participants were recruited in two French Expert Centers for ASD, coordinated by the Fundation FondaMental. Psychiatric comorbidities, anxiety disorders and depression were screened with standard tools (Liebowitz social anxiety scale, Hamilton Depression and Anxiety Rating Scale) and correlated to autistic features and social skills assessed with the social responsiveness scale 2 (SRS-2) and the repetitive behavior scale (RBS-R). We performed bivariate analysis between the social anxiety level and the scores measured with different clinical scales. We then adjusted the observed relationships with the alterations of SRS-2 and RBS-R scores.

**Results:**

After adjustment, the level of social anxiety appeared as significantly associated with alterations in social reciprocity and particularly with the SRS-2 “social communication” and “social motivation” sub-scores, but not with RBS-R score.

**Conclusions:**

We confirm previous reports showing that individuals with ASD are at high risk for specific anxiety disorders. In particular, high levels of impairments in social motivation and social communication (SRS-2) are indicative of comorbid disorders namely, social anxiety and ASD. Our findings clearly inform diagnostic assessment in ASD and stress the need to take comorbid anxiety disorders into consideration to improve treatment of ASD. To further clarify the impact of social anxiety on social competences and socio-adaptive handicap, longitudinal studies and cluster analysis will be needed in the future.

## Introduction

The prevalence of co-occurring psychiatric disorders in Autism Spectrum Disorder (ASD) is estimated to be close to 70% (1). According to Leyfer et al. (2) as cited by Vaillancourt et al. (3), about 72% of children with ASD have at least one other psychiatric disorder, frequently internalizing problems, such as depression and anxiety, and externalizing problems, such as hyperactivity or behavioral difficulties (4).

Anxiety disorders are mainly represented among Typically Developmental Children and Adolescents (TDCA) with estimated rates around 16% (5) and particularly social anxiety disorder with an estimated prevalence of 7 to 13% of TDCA (6). Even if there is now considerable evidence that children and adolescents with ASD have an increased risk of developing anxiety and anxiety disorders, it is still not clear which of the specific DSM-V anxiety disorders occurs more frequently in this population and what are their prevalence.

The level of social anxiety among children and adolescents with ASD without intellectual disability is estimated to be higher than for typical adolescents (7). It can reach up to 49% in a sample of high-functioning adolescents with ASD (8). The prevalence is usually estimated to be close to 17% of individuals with ASD as described in a Meta-analysis based on 31 publications (9). The disparities in prevalence observed across studies may be attributable to differences in sampling and selection criteria (e.g., epidemiological vs clinical samples), methods of assessment (e.g., self- vs. clinician or parental-rated measures, or use of one vs. multiple measures), diagnostic overshadowing (whereby co-morbid symptoms are wrongly attributed to ASD alone), or impairments in cognitive functioning (e.g., in introspection). Moreover, it is often difficult for individuals with ASD to describe their internal states. Thus, measuring anxiety is always a challenge in this population (10). In addition, we have yet no French data to describe the prevalence of anxiety disorders in population of children and adolescents with ASD.

The use of the Diagnostic and Statistical Manual of Mental Disorders - V (DSM-V) criteria (11) or of the International Classification of Diseases (ICD-10) (12) may underestimate the association between ASD and SA. Diagnoses seem more precise by using multimodal assessment methods, based on evaluation questionnaires, completed by clinicians and parents (13, 14). Indeed, the relationship between core symptoms of ASD and social anxiety symptoms is complex and often challenges clinicians. Although they are two distinct categorical disorders, they share similar dimensional symptoms and the degree of overlap may lead to a misinterpretation of symptoms. Both share impairments in social interactions or for example reduced eye contact (15). Therefore, part of ASD features heterogeneity may be related to comorbid social anxiety symptoms intensity.

The literature supports the idea that there are links between ASD features and social anxiety and that characteristic social skills impairments in ASD may contribute to the development of social anxiety and negatively impact social experiences. More generally, it has been suggested that a lack of social skills may lead to negative reactions from others, which promote negative beliefs and avoidant behaviors in social situations in the general population (16). Only few studies have examined the effect of anxiety disorders and particularly social anxiety on social functioning in typically developing children. Spence et al. first investigate in 1999, then, in 2004, the impact of this specific anxiety disorder and the link with social skills of children. The authors found that in children without ASD and compared to non-anxious children, children with Social Anxiety Disorder have been found to have poorer social skills on behavioral assessments in the laboratory and in school (17, 18). Their parents reported less assertive and responsible social skills and researchers observed fewer initiation and social interaction.

Recent studies examined specific associations between SA and social functioning in children and adolescents with ASD by using various different scales (SIB-R: Scales of Independent Behavior-Revised, BASC- SRS: Behavior Assessment System for Children Self and parental Report; self-report measures of social difficulties; ADI-R sub-scores…) but these studies lacked diagnostic anxiety measures. Results of the first review in 2009 didn’t conclude to an association between social anxiety and poorer social functioning in ASD (19), but other more recent studies showed that poorer social functioning in ASD correlates with elevated SA scores (20–23). Significant relationships between ASD severity and the parental-rated Revised Child Anxiety and Depression Scale (24), the parental-rated K-SADS-PL (25) or clinician-rated anxiety symptoms with the MINI-plus (26) was described. Five studies in adults with ASD (26–30) unlighted associations between self-reported social anxiety on the Liebowitz Social Anxiety Scale LSAS and socio communicative impairments.

More precisely, a recent systematic review shown that in individuals with ASD, SA was associated with poorer social skills, and in particular reduced the social motivation for social behaviors. Authors suggest that inherent social communication impairments in ASD can influence the number and quality of social situation and relationship, and lead to diminished social motivation. Poorer social motivation assessed *via* both various type of measures, self-, and informant-rating scales, was associated with increased levels of social anxiety (10). These findings seem to be replicated between the different studies, despite the wide range and type of measures of ASD symptoms and SA symptoms used in child, adolescent and adult samples (self or informant rating scales) that precluded formal meta-analysis.

Side to the social skills and social motivation deficit hypothesis, some argued that in ASD, stereotyped and repetitive behaviors or conversations may lead to stigmatization and may increase susceptibility to social adversity or bullying and victimization (31), thereby contributing to social withdrawal and segregation (32). One study reported significant relationship between higher total anxiety score and increased stereotyped behaviors, using the parent-reported CASi-anxiety (33).

The overlapping symptomatology and altered presentations of symptoms of anxiety contribute to the challenges of assessment and treatment of this condition. They require a multidisciplinary and specific approach as comorbid symptoms are wrongly attributed to ASD. Because of the great impact on the course of the disorder, recognizing anxiety and treating it properly is particularly important for the wellbeing of these patients. Moreover, while untreated comorbid anxiety has been associated with the development of depression, aggression, and self-injury in ASD (34), early recognition and treatment may convey better prognosis for those patients.

The main aims of our study were: (1) to assess the level of social anxiety and the prevalence of psychiatric comorbidities in a French cohort of children and adolescents with ASD compared to a control group, (2) to explore the association of social anxiety symptoms with demographic criteria, symptoms of depressions and anxiety, social functioning and specially social motivation, repetitive behaviors and adaptative behaviors.

## Methods

### Participants

79 children and adolescents from the French InFoR Autism cohort were selected for this study. They were recruited from March 2013 to February 2016 in two French Expert Centers on ASD in Bordeaux and Paris. The evaluations at inclusion of children and adolescents (aged 6 to 18 years) with a formal diagnosis of ASD according to DSM-V criteria (Diagnostic and Statistical Manual of Mental V) were taken into account (11). An Autism Diagnostic Interview - Revised (ADI-R) (35) or an Autism Diagnostic Observation Schedule (ADOS) (36) was also performed. In addition, 28 matched control participants (without ASD) were also included. The non-inclusion criteria for all subjects were an intelligence quotient (IQ) < 50 or the presence of a previously diagnosed genetic disorder.

Parents of children and adolescents have all filled a written consent form. The InFoR Autism study has been approved by a Paris’ regional ethics review board and the national commission on informatics and liberty. This study is part of clinical trial C07-33 sponsored by Inserm. It was granted approval by local Ethics Committee or “Comité de Protection des Personnes” on 2008 November 19th, authorized by the French authorities (AFSSAPS B80738-70 on 2008 August 11th), and registered in a public trials registry (NCT02628808).

### Materials and Procedure

The INFOR Autism Study is a multicentric study that involved Children, Adolescents, and Adults with ASD. In each center, a psychiatrist and a clinical psychologist performed the different explorations and help parents to understand and complete the various different scales. The clinical assessments attempted to be based on scales that have been validated in French population, available in 2013 and to adapt them to various ages samples. Parental assessment of symptoms of anxiety and depression was chosen to explore clinical features of anxiety and depression, in 2013 first because no self-report scale had been validated at that time in French children and adolescents and even less in children and adolescents with ASD. Then, secondly because self-report scales were under debate in this population. In a review of 40 studies of youth with anxiety and an ASD (19) the authors urged researchers to examine the applicability of traditional self-measures of childhood anxiety to the ASD population. Children with an ASD may have difficulty completing self-assessments and rating scales of their own levels and experiences with anxiety; therefore, authors advised interviewing caregivers that may help to provide additional information regarding the severity of the anxiety that the children may not be able to express (37). Parents were trained by clinicians involved in the project to answer the many different clinical and neuropsychological scales and items of scales that could have not always been adapted at live context of the child. Clinicians took time to explain and found various examples suitable for children that allows parents answering various emotional, social and communicational items.

ASD features were explored using the French translation of the social reciprocity scale SRS-2 (Social Responsiveness Scale-2) (38), and the French translation of the Repetitive Behavior Scale - Revised (RBS-R) (39). The raw scores of SRS-2 recommended for research (38, 40), were used to generate five sub-scores for the five symptoms domains: social awareness, social cognition, social communication, social motivation and restricted interests, and repetitive behavior, in order to measure deficits in social skills associated with ASD. The six sub-scores of the RBS-R that explore the breadth of restrictive interests and repetitive behavior in autism (stereotyped behavior, self-injurious behavior, compulsive behavior, routine behavior, sameness behavior and restricted behavior) were recorded.

Symptoms of anxiety and social anxiety were assessed with the French translations of the Hamilton Anxiety Rating Scale (HARS) (41) and of the Liebowitz Social Anxiety Scale (LSAS) (42–44). The Liebowitz Social Anxiety Scale (LSAS) is one of the most popular measures of social anxiety in adults in general population and clinical sample, in addition, the LSAS showed a good cross-cultural consistency, corroborating the psychometric qualities found in the first earlier samples.The LSAS evaluates fear and avoidance behaviors in social interaction situations and performance situations using Likert scales of 0 to 3. It is a 24-item report questionnaire measuring social anxiety in the general population that has been translated in French and were filled by the parents. The scale requires participants to imagine being in different social situations and asks how much fear they would experience and how much they would avoid the situation. The LSAS has demonstrated good test–retest reliability and discriminant validity (45). The overall gross total score was calculated by summing the total fear and total avoidance scores and was used for the analyses. This scale has been adapted and validated into French (43, 44), in this adult form, available in 2013. The LSAS has been adapted at a later stage for clinical assessment of children and adolescents (LSAS-CA) (46) with a self-ratting English language version, but the French translation was conducted and validated only in 2014 and only in a general Belgian Adolescents population older than 14 years old (self-ratter form) (47) and was not available at the beginning of INFOR Autism project that started in 2013. Parents had the instructions to question their child for all social situations listed in the scale and clinicians help then to give examples of different contexts to explain and explore the social fear and avoidance (e.g., social situation at school replaced social professional situations- item 5, 6, 20, 16; or birthday party replaced adult social meeting (item 23), contact with vendors were illustrated if needed by situation of contestation of food in canteen staff ect…).

Symptoms of depression were explored with the French version of the Hamilton Depression Rating Scale (HDRS) (48) also filled by parents, and lifetime psychiatric diagnostics performed with the French version of the Kiddie - Schedule for Affective Disorders and Schizophrenia for School -Age Children – Epidemiological version (K-SADS-e) (49).

The Vineland Adaptive Behavior Scale II (50) was used as it measures adaptive skills by examining 3 areas: communication, daily living skills and socialization, to evaluate the impact on socio-adaptive behaviors. The dimension of motor skills was not selected as a variable of interest in the InFoR Autism study.

To assess the verbal functioning of the children, which may interfere with the social functioning, the children Communication Checklist CC2 (51) was used, evaluating the general communication dimension.

### Statistical Analysis

We first performed a descriptive analysis of the 79 subjects with ASD and the 28 controls with mean (m) and standard deviation (sd) for continuous variables and percentage (%) and count (n) for discrete variables. A comparison between the two groups was also performed using a Pearson’s Chi-squared test with an estimate of the p-value according to a Monte Carlo method of 1000 iterations for discrete variables, and a Student’s T-test with Welch correction or Wilcoxon-Mann-Whitney’s (non-parametric) tests for continuous variables. Next, we performed bivariate analysis between the level of social anxiety of the participants with ASD and the various scores of the different clinical scales. Pearson or Spearman (non-parametric) correlation tests and estimation of coefficient of determination (R^2^) were applied for pairs of quantitative variables. Student’s T-test with Welch correction or Wilcoxon-Mann-Whitney’s tests (non-parametric) were applied in complement of punctual biserial correlation coefficient (r_pb_) for pairs of a qualitative and a quantitative variable. Parametric or non-parametric tests were chosen depending on the normality of analysed variables. Distributions were considered as normal if Shapiro-Wilk, Anderson-Darling and Jarque-Bera’s tests were not significant.

Then, we adjusted the observed relationships on the alterations of social reciprocity (SRS-2 score) and restricted interests and repetitive behaviors (RBS-R score) by applying a partial correlation. The partial version of the Pearson or Spearman’s correlation tests (non-parametric) and an estimate of the partial coefficient of determination were applied.

All analysis using the LSAS “fear” sub-score and “avoidance” sub-score separately are available in supplementary materials.

All analyses were performed in available cases (data available for the pair of variables or the triplet of variables analyzed) using the programming language R version 3.5.1 (52). Graphics were created using additional packages ggplot2 and scatterplot3d (53, 54). Packages versions were fixed at July 2^nd^ 2018 with the checkpoint package (55). Significant level was fixed to 5% and p-value was two-tailed.

## Results

### Population Description

On 79 subjects with ASD 40 were adolescents (50.66%) and the mean age was 11.51 years (sd = 3.13). Almost all were men (89.87%) and most had high functioning (79.75%) with IQ>70. 72% of participants reported anxiety disorders, 25.8% of them presented criteria for Social Anxiety diagnosis, and 18.9% reported Agoraphobia without panic disorder. 18,9% of ASD participants reported depression and 55.7% criteria for ADHD, based on KIDDIE-SADS-PL. The mean social anxiety level assessed using the LSAS was moderate with an important variability (m = 53.18, sd = 33.61). Means levels of anxiety and depression were pretty low, with respectively m = 2.9 (sd = 4.98) and m = 1.63 (sd = 2.95). In addition, 15 persons on 79 (18.99%) reported a depressive disorder in their lifetime and 1 (1.27%) a recurrent depressive disorder. Concerning autistic symptoms, the social reciprocity dimension represented by the SRS-2 raw score was highly altered (m = 178.08, sd = 46.78) and the repetitive behavior and restricted interest dimension represented by the RBS-R score was moderately impacted (m = 25.24, sd = 16.66).

Control subjects and patients with ASD were comparable in age and levels of depressive and anxious symptoms assessed by the HDRS and the HARS, but not on sex ratio. Moreover, there was a significant difference in social anxiety level between the two groups (p < 0.001). About the 2 autistic domains symptoms, as expected the group with ASD scored significantly higher than the control group on the SRS-2 (p < 0.001) and the RBS-R (p < 0.001). In addition, the adaptive skills of subjects with ASD was significantly more impaired in all domains assessed using the VABS II (p < 0.001), as the communication skills assessed using the CC2 (p < 0.001).

**Table 1** shows the descriptive statistics for each of the demographic and clinical characteristics for the autistic group and the control group.

**Table 1:** Population.

	Subjects with ASD			Controls			p
	N	%	n	N	%	n	
Male	79	89.87	71	28	60.71	17	0.507
Adolescent	79	50.66	40	28	42.86	12	**0.002**
High functioning (IQ > 70)	79	79.75	63	28	100	28	NA
Social anxiey disorder	58	25.86	15	5	0	0	NA
Depressive disorder	79	18.99	15	28	0	0	NA
Recurrent depressive disorder	79	1.27	1	28	0	0	NA
Panic disorder with agoraphobia	58	0	0	5	0	0	NA
Panic disorder without agoraphobia	58	1.72	1	5	20	1	NA
Agoraphobia without panic disorder	58	18.97	11	5	0	0	NA
Obsessive compulsive disorder	58	12.07	7	5	0	0	NA
	**N**	**m**	**sd**	**N**	**m**	**sd**	**p**
Age	79	11.51	3.13	28	10.46	3.62	0.148
LSAS	60	53.18	33.61	24	14.92	14.98	**<0.001**
LSAS anxiety	60	25.57	17.09	24	4.58	9.77	**<0.001**
LSAS avoidance	63	27.97	17.14	24	10.33	6.43	**<0.001**
HARS	78	2.9	4.98	28	0.93	2.12	0.111
HDRS	79	1.63	2.95	28	0.89	2.48	0.315
SRS 2 Total	73	178.08	46.78	25	35.72	26.04	**<0.001**
SRS 2 Restricted interests and repetitive behavior	73	19.45	5.99	25	1.64	1.55	**<0.001**
SRS 2 Social communication	73	33.18	9.85	25	6.16	5.73	**<0.001**
SRS 2 Social awareness	73	11.23	3.76	25	3.84	2.3	**<0.001**
SRS 2 Social cognition	73	18.42	5.27	25	2.88	3.23	**<0.001**
SRS 2 Social motivation	73	16.48	6.76	25	4.16	3.66	**<0.001**
RBS-R Total	67	25.24	16.66	25	2	2.87	**<0.001**
RBS-R Sterotyped behavior	73	4.15	3.6	25	0.12	0.44	**<0.001**
RBS-R Self-injurious behavior	73	1.38	2.14	25	0.08	0.4	**<0.001**
RBS-R Compulsive behavior	70	3.69	3.75	25	0.36	0.64	**<0.001**
RBS-R Routine behavior	69	4.64	4.02	25	0.12	0.33	**<0.001**
RBS-R Sameness behavior	70	7.87	6.17	25	0.8	1.22	**<0.001**
RBS-R Restricted behavior	71	3.85	2.54	25	0.52	1.08	**<0.001**
VABS II total	74	222.99	40.42	26	326.19	31.92	**<0.001**
VABS II Communication	76	75.7	12.58	27	106.86	11.01	**<0.001**
VABS II Daily living skills	75	73.73	14.46	27	102.15	9.31	**<0.001**
VABS II Socialization	75	69.67	14.32	27	112.37	9.12	**<0.001**
CC2 general communication	66	30.85	13.53	24	88.42	12.89	**<0.001**

N, sample size for variable; %, percentage; n, number of subjects with the characteristic; m, mean; sd, standard deviation; p, p-value; NA, not applicable. Bold values denote statistical significance at the p < 0.05 level.

LSAS, Liebowitz Social Anxiety Scale; HARS, Hamilton Anxiety Rating Scale; HDRS, Hamilton Depression Rating Scale; SRS 2, Social Responsiveness Scale 2; RBS-R, Repetitive Behavior Scale – Revised; VABS II, Vineland Adaptive Behavior Scale II; CC2, Children Communication Checklist.

### Dimensions Associated With Social Anxiety

The results presented in **Table 2** showed no significant association between LSAS score and age (ρ = 0.23, R^2^ = 0.04, p = 0.076) or age category (r_bp_ = 0.24, η^2^ = 0.06, p = 0.061), as well as IQ level (r_bp_ = -0.17, η^2^ = 0.03, p = 0.215). Social anxiety level was significantly associated with anxious symptoms level represented by HARS score (ρ = 0.29, R^2^ = 0.08, p = 0.022) but not with depressive symptoms level represented by HDRS score (ρ = 0.11, R^2^ = 0.01, p = 0.399).

**Table 2:** Correlations between Liebowitz social anxiety scale and demographical and clinical factors in ASD.

	N	r_pb_	p	η^2^
Children → Adolescents	60	0.24*	0.061	0.06
Low functioning → High functioning	60	-0.17*	0.215	0.03
	**N**	**ρ/r**	**p**	**R^2^**
Age	60	0.23*	0.076	0.04
HARS	60	0.29*	**0.022**	0.08
HDRS	59	0.11*	0.399	0.01
SRS 2 Total	59	0.49*	**<0.001**	0.23
SRS 2 Restricted interests and repetitive behavior	59	0.32*	**0.013**	0.11
SRS 2 Social communication	59	0.42*	**<0.001**	0.16
SRS 2 Social awareness	59	-0.05*	0.731	0
SRS 2 Social cognition	59	0.31*	**0.015**	0.11
SRS 2 Social motivation	59	0.62*	**<0.001**	0.39
RBS-R Total	55	0.39*	**0.003**	0.15
RBS-R Sterotyped behavior	60	0.24*	0.066	0.08
RBS-R Self-injurious behavior	60	0.28*	**0.033**	0.03
RBS-R Compulsive behavior	58	0.25*	0.055	0.08
RBS-R Routine behavior	57	0.21*	0.125	0.03
RBS-R Sameness behavior	57	0.45*	**<0.001**	0.18
RBS-R Restricted behavior	58	0.19*	0.144	0.04
VABS II total	59	0.01*	0.938	0
VABS II Communication	60	0.08*	0.556	0.02
VABS II Daily living skills	60	0.09*	0.472	0.02
VABS II Socialization	59	-0.05*	0.706	0
CC2 general communication	54	-0.24*	0.074	0.07

N, sample size for variable; r_pb_, punctual biserial correlation coefficient; p, p-value; η^2^, ratio correlation coefficient; ρ, coefficient of correlation of Spearman; r, coefficient of correlation of Pearson; R^2^, coefficient of determination; *, non-parametric test. Bold values denote statistical significance at the p < 0.05 level.

HARS, Hamilton Anxiety Rating Scale; HDRS, Hamilton Depression Rating Scale; SRS 2, Social Responsiveness Scale 2; RBS-R, Repetitive Behavior Scale – Revised, VABS II, Vineland Adaptive Behavior Scale II; CC2, Children Communication Checklist.

Autistic symptoms were associated with social anxiety level in a complex manner. LSAS raw score was positively and significantly linked with SRS-2 total score (ρ = 0.49, R^2^ = 0.23, p < 0.001). All SRS-2 sub-scores, except social awareness, were significantly associated with social anxiety and especially social motivation (ρ = 0.62, R^2^ = 0.39, p < 0.001). The RBS-R total score was significantly linked with the LSAS score (ρ = 0.39, R^2^ = 0.15, p = 0.003) The subcategory “self-injuring behavior” shows a moderate correlation (ρ = 0.28, R2 = 0.03, p = 0.033) and “sameness behavior” a strong one (ρ = 0.45, R^2^ = 0.18, p < 0.001).

**Figure 1:**
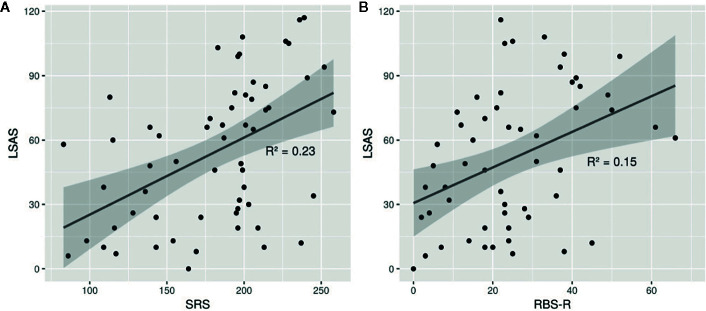
Point clouds representing the relationship between LSAS and SRS-2 total raw score/RBS-R total score. In **(A)**, we could observe a positive relationship between the LSAS score and the SRS-2 score with a moderate correlation represented by a coefficient of determination R^2^ = 0.23. In **(B)**, we could observe a weaker relationship between the LSAS score and the RBS-R score with a moderate correlation represented by a coefficient of determination R^2^ = 0.15. LSAS, Liebowitz Social Anxiety Scale; SRS-2, Social Responsibility Scale - 2; RBS-R, Repetitive Behavior Scale – Revised.

**Figure 1** illustrates the relationship between social anxiety and social skills impairments (SRS scale) and between social anxiety and restricted interests and repetitive behaviors (RBS-R scale). There is a positive relationship with a moderate correlation represented by a coefficient of determination R^2^ = 0.23 between LSAS scores and SRS-2. This correlation is weaker between the LSAS scores and the RBS-R (R^2^ = 0.15).

High functioning participants had higher standardized scores for all dimensions of the Vineland scale that explored socio-adaptative dimensions of the participants (N = 60) compared to low functioning participants (N=14-16, depending on the dimension) (all p < 0.011). A summary of the results can be found in **Table 3**. Moreover, there was no significant association between Intellectual functioning based on total IQ index nor socio-adaptive behaviors assessed by the VABS II and social anxiety that can be highlighted in our sample (respectively r_pb_ = 0.17, η^2^ = 0.03, p = 0.215; ρ = 0.01, R^2^ = 0, p = 0.938). Participants with lower IQ were participants with more socio-adaptative impairments.

**Table 3:** Descriptive statistics for High and Low functioning patients.

	active Ns*	m*	sd*	p
Communication	60/16	79.1/63	10.9/10.1	**<0.001**
Daily living skills	60/15	75.8/65.3	14.5/11.1	**0.011**
Socialization	60/15	72.1/60	14/11.4	**0.003**
Adaptive behavior composite	60/14	73.3/61.4	9.9/9.2	**<0.001**

N, sample size for variable; m, mean; sd, standard deviation; p, p-value. Bold values denote statistical significance at the p < 0.05 level. * = High/Low functioning.

No association was found between the general communication assessed using the CC2 and the LSAS score.

### Adjusted Analysis

In order to take into account the severity of symptoms in the two main domains impaired in ASD and try to isolate sub-groups of patients expressing Social Anxiety, we performed a trivariate analysis to highlight associations after adjustment to the severity of social impairments and repetitive behaviors symptoms assed either by SRS-2 and RBS-R scores. We first applied a partial correlation using the LSAS raw score on the one hand and the demographic and clinical variables, adjusted on the SRS-2 scores then on the RBS-R scores (see **Table 4**).

**Table 4:** Correlations between Liebowitz social anxiety scale and demographical and clinical aspects after adjustments.

Adjusted on: SRS 2	N	pρ/pr	p	pR^2^
Age	59	0.31*	**0.019**	0.08
HARS	59	0.25*	0.057	0.04
HDRS	58	0.04*	0.764	0
RBS-R Total	54	0.17*	0.212	0.03
RBS-R Stereotyped behavior	59	0.08*	0.571	0.02
RBS-R Self-injurious behavior	59	0.19*	0.164	0.01
RBS-R Compulsive behavior	57	0.14*	0.294	0.04
RBS-R Routine behavior	56	-0.01*	0.929	0
RBS-R Sameness behavior	56	0.29*	**0.03**	0.07
RBS-R Restricted behavior	57	-0.03*	0.825	0
VABS II total	58	0.09*	0.491	0.01
VABS II Communication	59	0.15*	0.248	0.03
VABS II Daily living skills	59	0.18*	0.176	0.03
VABS II Socialization	58	0.07*	0.583	0
CC2 general communication	53	0.02*	0.865	0.01
**Adjusted on: RBS-R**	**N**	**pρ/pr**	**p**	**pR^2^**
Age	55	0.34*	**0.012**	0.09
HARS	55	0.29*	**0.033**	0.08
HDRS	54	0.18*	0.209	0.02
SRS 2 Total	54	0.36*	**0.008**	0.12
SRS 2 Restricted interests and repetitive behavior	54	0.06*	0.689	0
SRS 2 Social communication	54	0.33*	**0.016**	0.08
SRS 2 Social awareness	54	-0.12*	0.378	0
SRS 2 Social cognition	54	0.22*	0.117	0.05
SRS 2 Social motivation	54	0.59*	**< 0.001**	0.35
VABS II total	54	0.08*	0.578	0.01
VABS II Communication	55	0.14*	0.326	0.03
VABS II Daily living skills	55	0.16*	0.245	0.03
VABS II Socialization	54	0.08*	0.562	0
CC2 general communication	50	-0.14*	0.338	-0.21

N, sample size for variable; p, p-value; pρ, partial coefficient of correlation of Spearman; pr, partial coefficient of correlation of Pearson; pR^2^, partial coefficient of determination; *, non-parametric test. Bold values denote statistical significance at the p < 0.05 level.

HARS, Hamilton Anxiety Rating Scale; HDRS, Hamilton Depression Rating Scale; SRS 2, Social Responsiveness Scale 2; RBS-R, Repetitive Behavior Scale – Revised; VABS II, Vineland Adaptive Behavior Scale II; CC2, Children Communication Checklist.

When adjusted on the SRS-2 score the association between social anxiety level and age become significant (pρ = 0.31, pR^2^ = 0.08, p = 0.019) but not the association between the HARS score and the social anxiety level (pρ = 0.25, pR^2^ = 0.04, p = 0.057), neither between the LSAS score and RBS-R scores (total and sub-scores), except for the “sameness behavior” sub-score (pρ = 0.29, pR^2^ = 0.07, p = 0.03).

When adjusted on the RBS-R score, the association between social anxiety level and age is significant (pρ = 0.34, pR^2^ = 0.09, p = 0.012), and the association with HARS score remained significant (pρ = 0.29, pR^2^ = 0.08, p = 0.033) as expected. Statistically significant associations were also found between social anxiety level and the SRS-2 total score (pρ = 0.36, pR^2^ = 0.12, p = 0.008) the social communication sub-score (pρ = 0.33, pR^2^ = 0.08, p = 0.016) and the social motivation sub-score with a strong positive correlation (pρ = 0.59, pR^2^ = 0.35, p < 0.001).

**Figure 2:**
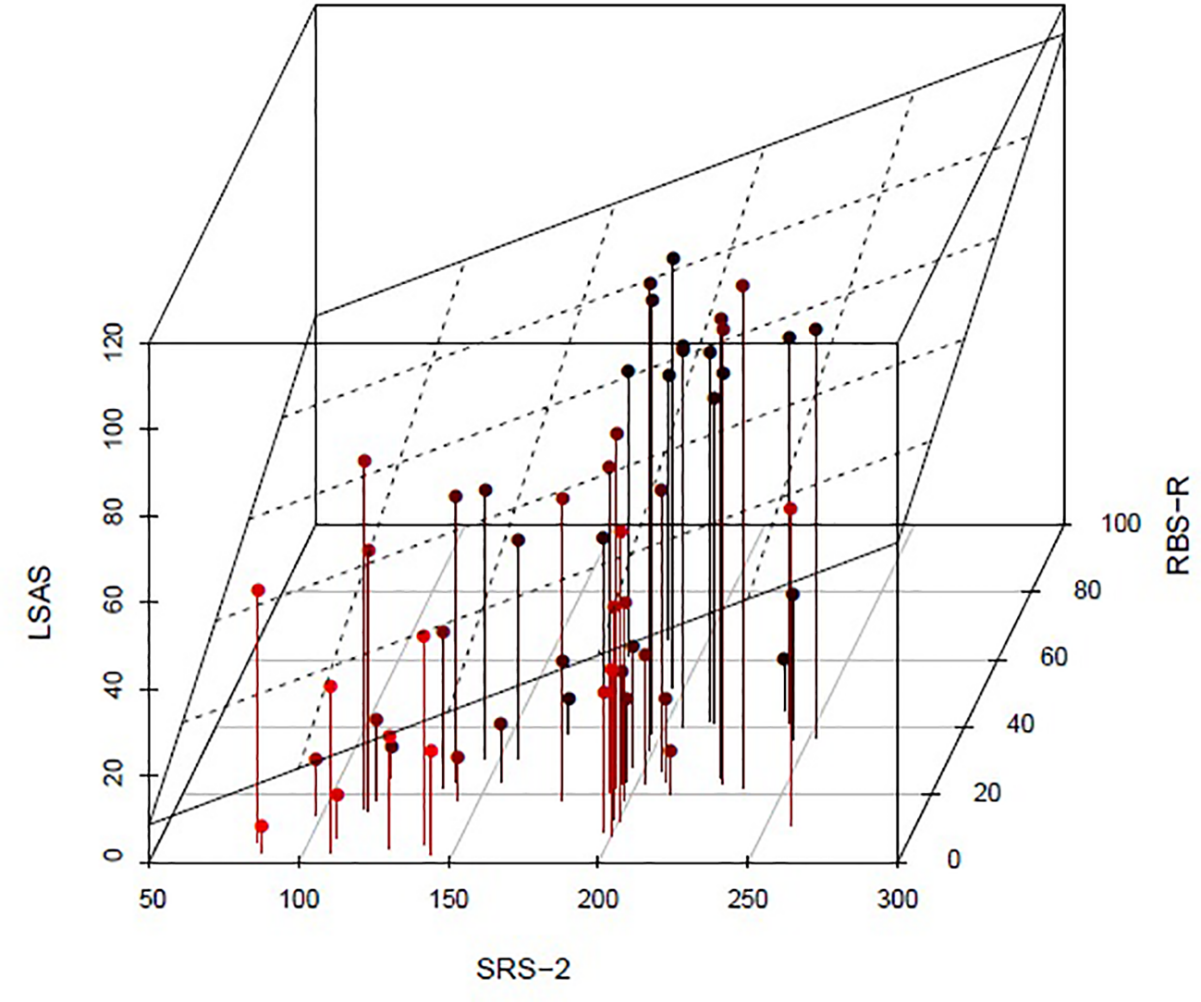
Relationship between LSAS, SRS-2, and RBS-R scores after adjustments with a 3 dimensions point cloud. The linear regression plan of the LSAS was represented. The dots are colored in black red gradient. The darker they are, the higher the RBS-R score is. On the frontal face of the square, the intersection with the regression plan with a strong positive angle shows a strong relationship between the LSAS and the SRS-2 scores even after adjustment on the RBS-R score. On the right lateral face, the intersection with the regression plan with a low positive angle shows a weaker relationship between the LSAS and the RBS-R scores after adjustment on the SRS-2 score. LSAS, Liebowitz Social Anxiety Scale; SRS-2, Social Responsibility Scale - 2; RBS-R, Repetitive Behavior Scale – Revised.

**Figure 2** illustrates the relationship between social anxiety and SRS-2 and RBS-R scores after adjustments. On the frontal face of the square, the intersection with the regression plan shows a strong relationship between the LSAS and the SRS-2 scores even after adjustment on the RBS-R score. On the right lateral face, the intersection with the regression plan shows a weaker relationship between the LSAS and the RBS-R scores after adjustment on the SRS-2 score.

No association between social anxiety symptoms and socio-adaptive behaviors assessed by the VABS II was found despite adjustment on the severity of clinical symptoms of social impairments and of repetitive behaviors in our sample.

## Discussion

This study demonstrates that Social Anxiety constitute a separate condition, comorbid of the core symptoms presented by children and adolescents with ASD which means that Social Anxiety need to be specifically assessed and treated. In addition, we found a prevalence of 25.6% for social anxiety disorder in French children and adolescents with ASD without mental retardation. This high prevalence replicates the previously reported prevalence of 11.7% to 29.2% (9, 56, 57). As hypothetized, socio-adaptive skills were specifically altered among children with ASD in comparison to controls (58), but were not associated to symptoms of social anxiety in autism. We also observed that the level of social anxiety increased with age in individuals with ASD, as already been reported (9).

Social anxiety is positively associated with social functioning deficits including social communication and social interactions impairments in previous studies (20, 59). Consistent to prediction, we showed a significant association between intensity of social anxiety and alterations in social reciprocity and social skills, related to a more marked alteration of social communication and social motivation among these patients. The strongest association was found with the social motivation sub-score. Social motivation refers to the interest in adopting social interpersonal behavior and starting social interactions with others (38). Corbett et al. (60) have already explored the association between social motivation and anxiety by measuring cortisol reactivity during a social task in children with ASD. They reported that the children with ASD who expressed the highest levels of cortisol reactivity, considered as a marker of anxiety, during play sequences, reported less social motivation. In particular, studies have described an association between social anxiety disorder among individuals with ASD and the social motivation scale of the SRS-2 in adults, showing that low social motivation was associated with increased social anxiety (61) and that both may be reinforced by avoidance of social situations. Here, we found that this association already reported in adults, exist early during the developmental course of the Autism Spectrum Disorders. Both socio-communication and motivation impairments and social anxiety seem to participate to social skills deficit observed in a sub-group of ASD individuals (10, 14, 62). While it may be partly due to overlapping factors assessing social fears, children with ASD and social anxiety may show a more negative assessment of the social situation resulting in social fears and less motivation to engage social interactions.

In addition, higher level of stereotyped and restricted behaviors was described in presence of social anxiety symptoms (22) which is partially confirmed in our study. The function of stereotyped behaviors in ASD has often been questioned, and it has been suggested that they may correspond to a strategy to reduce anxiety (59). This role would be attributed more particularly to insistence on sameness (63). In our study, insistence on sameness sub-score was associated with social anxiety in line with the results of a recent study (64).

The potential mediating effects of social motivation between anxiety and repetitive behaviors and restricted interests, and particularly insistence on sameness in children with ASD have been explored in a previous study (65). Social anxiety was associated with social motivation and insistence on sameness, and then the relationship between anxiety and sameness behaviors was partially reported to be mediated by social motivation. Our results are therefore consistent with these data while confirming more particularly the implication of social anxiety on these clinical manifestations of ASD (mediated by social motivation). These results suggest that treating social anxiety symptoms may reduce both social anxiety deficits and insistence on sameness in children and adolescents with ASD. It may also be worth to include in the future a comparison group of non ASD subjects with characterized Social Anxiety, recorded with various measure of social anxiety but also social motivation and socio communication skills (self and informant reports) in order to establish the direction of the association between social communication deficits and social anxiety and to determine if the anxiety shown by children with ASD is rather a disorder of a way to adapt to their social difficulties.

As anxiety is a multifaceted construct involving behaviors, cognitions, affect, and physiological arousal, the assessment method of Social Anxiety in Autism needs to be discussed. A limitation to consider is related to the collection procedure and the scales used to assess clinical emotional symptoms. Self-reporting, informant reporting, clinician-assessed measures, or even the use of multiple measures do indeed exhibit “variance bias”, particularly in our sample. The parents were the informants for main clinical and neuropsychological scales in INFOR Project. A measuring biases must be taken in account in results recorded in such multicentric projects to discuss hypothesis and conclusions. Since the end of INFOR project, translation and adaptation of the different scales for children and Adolescents are now available and further studies, employing qualitative and quantitative French language validated scales and multi-assessment designs are needed to enhance description and understanding of causal, maintaining, and protective mechanisms for SA in ASD. In the screening of the association of SA and ASD, significant predictors of informant-ratings of SA include goal-directed behavior for negative emotions, impaired awareness of emotions, and social motivation. Authors suggest that negative self-image, or depression, might lead to more severe self-ratings for both ASD and SA in self-rating methods. Despite the difficulty for informants to accurately endorse cognitive and affective characteristics indicative of SA, results suggest that correlations between measures from the participant him-self may be inflated (10). Other authors suggested risk of underestimated measures of anxiety in the case of ASD due to the inherent communication impairments and “alexithymia” (i.e., poor emotional insight and difficulty describing feelings) and excluded traditional assessment of anxiety using self-report in ASD people (66, 67). More generally, how self-report questionnaires operate for individuals with ASD is yet to be definitively established. Spain et al. (10) systematic review recommend the use of multiple methods of assessment. Studies employing multiple methods of assessment, such as self-rated questionnaires, and clinician-administered interviews and biological measures (e.g., of anxiety) may aid with understanding discrepancies between these ratings (10). Our results need to be replicated using multiple assessments including biological measures of anxiety and of social motivation at various developmental ages in order to explore the direction of association between Social Anxiety and social skills impairments and the effect of social experiences.

Another limitation must be mentioned concerning the comparability of the two groups. First, differences are present for the sex ratio between the two groups. Indeed, there is a greater female representativeness in the control group (40% compared with 10% in the ASD group), while gender is known to be a factor involved when considering emotional expression in typical and atypical population. This must be taken into account for future studies. Moreover 20% of the ASD group had an IQ<70 (Low functioning ASD) and the control sample only included individuals with IQ>70 (High functioning ASD). As we observe no statistic differences between High Functioning patients (HF) vs Low Functioning (LF) in terms of interesting variable (LSAS mean: HF = 55.7, LF = 40.7, p = 0.23), we did not stratify participants, but these results must be taken with caution in regards to the low size of the LF group (N=16). ASDs are neurodevelopmental disorders that require age group considerations. Unfortunately, the sample size did not allow us to consider 2 strictly comparable subgroups (children and adolescents), due to the size of the control group, which was not strictly age- and sex-specifically matched if we separated children from adolescents. Our results need to be replicated separately for children and adolescents, in larger groups with a strictly matched control group, in order to confirm the significant differences that were found between the two overall groups of participants under 18 years of age that we considered here.

There is a growing interest for psychiatric comorbidities among population with ASD particularly children and adolescents (56, 68, 69), because of their massive and predictive functional and socio-economic impact. Although the literature brings ample evidences that social anxiety disorder is often associated with ASD, most studies in children and adolescents did not consider it separately from other anxiety disorders. Further research are needed in particular longitudinal studies in order to define the impact of social anxiety on social competencies and socio-adaptive behaviors with ages and the link with social motivation that could be specifically described in the field of ASD. There is a particular intricacy between the social dimension in ASD and social anxiety, and taking into account this entanglement could help refine clinical assessment in ASD and propose early interventions targeting social anxiety in this population in order to improve social motivation.

## Data Availability Statement

The datasets presented in this article are not readily available because of a confidentiality agreement set in the frame of a Research Collaboration Agreement; namely the "InFoR Autism" project between Institut Roche, Inserm, Inserm transfer and Fondation FondaMental. Requests to access the datasets should be directed to InFoR Autism Joint Steering Committee.

## Ethics Statement

The studies involving human participants were reviewed and approved by a Paris’ regional ethics review board and the national commission on informatics and liberty. This study is part of clinical trial C07-33 sponsored by Inserm. It was granted approval by local Ethics Committee or “Comité de Protection des Personnes” on 2008 November 19th, authorized by the French authorities (AFSSAPS B80738-70 on 2008 August 11th), and registered in a public trials registry (NCT02628808). Written informed consent to participate in this study was provided by the participants’ legal guardian/next of kin.

## Author Contributions

KB, FJ, AJ, and AA: conception, design, drafting of the work and analysis and interpretation of data. DU, CC, and LM: acquisition, analysis or interpretation of data for the work, and provide approval for publication of the content. M-MG, ML-L, ML, RD, and MB: revising the work critically and provide approval for publication of the content.

## Funding

This work has been co-ordinated by the Fondation FondaMental and achieved thanks to the following organisms and establishments: AP-HP, CHU Bordeaux, Hôpital Charles Perrens, Robert Debré et Henri Mondor’s CIC. It was financially supported in part by the Institut Roche, in part by the Investissements d’Avenir program managed by the ANR under reference ANR-11-IDEX-0004-02.

## Conflict of Interest

DU, CC, and LM are current full-time employees of F. Hoffmann-La Roche Ltd. and received support in form of salaries. ML-L is a full-time employee of Institut Roche and received support in form of salaries.

The remaining authors declare that the research was conducted in the absence of any commercial or financial relationships that could be construed as a potential conflict of interest.
