# Supplementary material for: Social Anxiety in Children and Adolescents With Autism Spectrum Disorders Contribute to Impairments in Social Communication and Social Motivation
Source: Front Psychiatry. 2020 Jul 24;11:710. doi: 10.3389/fpsyt.2020.00710 (PMC7393242; doi:10.3389/fpsyt.2020.00710)
Supplement: Supplementary file 1 [file DataSheet_1.pdf]

Supplementary material Table 1: Correlations between Liebowitz social anxiety sub-scales and demographical and clinical factors in ASD

	LSAS Fear score				LSAS Avoidance score			
	**N**	r_{pb}	**p**	η^2	**N**	r_{pb}	**p**	η^2
Children → Adolescents	60	0.24*	0.071	0.06	63	0.22*	**0.035**	0.071
Low functioning → High functioning	60	-0.17*	0.174	0.03	63	-0.16*	0.288	0.174
	**N**	**ρ/r**	**p**	R^2	**N**	**ρ/r**	**p**	R^2
Age	60	0.22*	0.096	0.03	63	0.28*	**0.025**	0.07
HARS	60	0.28*	**0.029**	0.06	63	0.29*	**0.023**	0.09
HDRS	59	0.12*	0.354	0.02	62	0.1*	0.419	0.02
SRS 2 Total	59	0.47*	**< 0.001**	0.22	62	0.47*	**< 0.001**	0.23
SRS 2 Restricted interests and repetitive behaviour	59	0.31*	**0.015**	0.11	17	0.34*	**0.007**	0.11
SRS 2 Social communication	59	0.42*	**< 0.001**	0.17	17	0.37*	**0.003**	0.15
SRS 2 Social awareness	59	-0.04*	0.762	0	17	-0.02*	0.895	0
SRS 2 Social cognition	59	0.29*	**0.024**	0.09	17	0.33*	**0.009**	0.13
SRS 2 Social motivation	59	0.6*	**< 0.001**	0.37	17	0.61*	**< 0.001**	0.39
RBS-R Total	55	0.34*	**0.012**	0.12	58	0.44*	**< 0.001**	0.18
RBS-R Sterotyped behaviour	60	0.24*	0.063	0.09	63	0.23*	0.072	0.06
RBS-R Self-injurious behaviour	60	0.27*	**0.034**	0.02	63	0.27*	**0.033**	0.03
RBS-R Compulsive behaviour	58	0.2*	0.137	0.06	61	0.33*	**0.008**	0.11
RBS-R Routine behaviour	57	0.19*	0.168	0.02	60	0.23*	0.078	0.03
RBS-R Sameness behaviour	57	0.42*	**0.001**	0.16	60	0.45*	**< 0.001**	0.2
RBS-R Restricted behaviour	58	0.13*	0.348	0.01	61	0.3*	**0.018**	0.09
VABS II total	59	-0.01*	0.969	0	62	0*	0.979	0
VABS II Communication	60	0.06*	0.648	0.01	63	0.04*	0.743	0.02

VABS II Daily living skills	60	0.11*	0.401	0.03	63	0.03*	0.805	0.01
VABS II Socialization	59	-0.07*	0.603	0	62	-0.02*	0.902	0

N: sample size for variable, r_{pb}: punctual biserial correlation coefficient, p : p-value, η^2: ratio correlation coefficient, ρ: coefficient of correlation of Spearman, r : coefficient of correlation of Pearson, R^2: coefficient of determination, * : non-parametric test

HARS: Hamilton Anxiety Rating Scale, HDRS: Hamilton Depression Rating Scale, SRS 2: Social Responsiveness Scale 2, RBS-R: Repetitive Behaviour Scale – Revised, VABS II: Vineland Adaptive Behavior Scale II

Supplementary material Table 2: Correlations between Liebowitz social anxiety sub-scales and demographical and clinical aspects after adjustments

Adjusted on: SRS 2	**LSAS Fear score**				**LSAS Avoidance score**			
	**N**	**pρ/pr**	**p**	**pR^2**	**N**	**pρ/pr**	**p**	**pR^2**
Age	59	0.31*	**0.019**	0.08	62	0.33*	**0.01**	0.09
HARS	59	0.25*	0.057	0.04	62	0.25*	0.059	0.05
HDRS	58	0.04*	0.764	0	61	0.03*	0.833	0
RBS-R Total	54	0.11*	0.416	0.02	62	0.24*	0.697	0.05
RBS-R Stereotyped behaviour	59	0.09*	0.525	0.02	62	0.05*	0.185	0.01
RBS-R Self-injurious behaviour	59	0.19*	0.162	0	60	0.17*	0.087	0.01
RBS-R Compulsive behaviour	57	0.08*	0.554	0.02	59	0.22*	0.948	0.06
RBS-R Routine behaviour	56	-0.03*	0.828	0	59	0.01*	**0.022**	0
RBS-R Sameness behaviour	56	0.26*	0.057	0.06	60	0.3*	0.434	0.09
RBS-R Restricted behaviour	57	-0.11*	0.41	0.02	62	0.01*	0.697	0.1
VABS II total	58	0.07*	0.594	0	61	0.11*	0.415	0.01
VABS II Communication	59	0.2*	0.141	0.04	62	0.14*	0.281	0.03
VABS II Daily living skills	58	0.05*	0.725	0	62	0.13*	0.303	0.02
VABS II Socialization	58	-0.17*	0.211	0.03	61	0.13*	0.335	0

Adjusted on: RBS-R	**LSAS Fear score**				**LSAS Avoidance score**			
	**N**	**pρ/pr**	**p**	**pR^2**	**N**	**pρ/pr**	**p**	**pR^2**
Age	55	0.34*	**0.012**	0.09	58	0.39*	**0.002**	0.12
HARS	55	0.29*	**0.033**	0.08	58	0.29*	0.027	0.1
HDRS	54	0.18*	0.209	0.02	57	0.16*	0.234	0.03
SRS 2 Total	54	0.37*	**0.007**	0.13	57	0.31*	**0.022**	0.11
SRS 2 Restricted interests and repetitive behaviour	54	0.09*	0.527	0.01	57	0.02*	0.867	0
SRS 2 Social communication	54	0.35*	**0.011**	0.1	57	0.24*	0.073	0.06
SRS 2 Social awareness	54	-0.1*	0.467	0	57	-0.14*	0.305	0
SRS 2 Social cognition	54	0.2*	0.149	0.04	57	0.21*	0.116	0.07
SRS 2 Social motivation	54	0.59*	**< 0.001**	0.33	57	0.55*	**< 0.001**	0.33
VABS II total	54	0.06*	0.651	0.01	57	0.08*	0.581	0.01
VABS II Communication	55	0.12*	0.394	0.02	58	0.1	0.458	0.03
VABS II Daily living skills	55	0.18*	0.183	0.04	58	0.11	0.416	0.02
VABS II Socialization	54	0.05*	0.709	0	57	0.13	0.352	0.01

N: sample size for variable, p: p-value, pρ: partial coefficient of correlation of Spearman, pr : partial coefficient of correlation of Pearson, pR^2: partial coefficient of determination, * : non-parametric test

HARS: Hamilton Anxiety Rating Scale, HDRS: Hamilton Depression Rating Scale, SRS 2: Social Responsiveness Scale 2, RBS-R: Repetitive Behaviour Scale – Revised, VABS II: Vineland Adaptive Behavior Scale II
